# Motivation to Move Out of the Community as a Moderator of Bullying Victimization and Delinquent Behavior: Comparing Non-Heterosexual/Cisgender and Heterosexual African American Adolescents in Chicago’s Southside

**DOI:** 10.3390/ijerph182412998

**Published:** 2021-12-09

**Authors:** Jun Sung Hong, Saijun Zhang, Rachel C. Garthe, Megan R. Hicks, Ellen W. deLara, Dexter R. Voisin

**Affiliations:** 1 School of Social Work, Wayne State University, Detroit, MI 48202, USA; gm5019@wayne.edu; 2 Department of Social Work, University of Mississippi, Oxford, MS 38677, USA; szhang9@olemiss.edu; 3 School of Social Work, University of Illinois at Urbana-Champaign, Urbana, IL 61801, USA; rcgarthe@illinois.edu; 4 Falk College of Sport & Human Dynamics, School of Social Work, Syracuse University, Syracuse, NY 13244, USA; edelara@syr.edu; 5 Factor-Inwentash Faculty of Social Work, University of Toronto, Toronto, ON M5S 1V4, Canada; dexter.voisin@utoronto.ca

**Keywords:** African American youth, sexual orientation, gender identity, hopefulness, delinquent behavior, bullying victimization

## Abstract

A growing body of research documents that bullying victimization is associated with delinquent behavior. There is an increasing need to better illuminate the factors that might moderate this relationship. This study examined whether the motivation to move out of low-resourced neighborhoods and sexual orientation/gender identity moderated the relationship between bullying victimization and delinquent behavior among a sample of 450 heterosexual and 91 non-heterosexual/cisgender African American youths. Measures considered were bullying victimization, delinquent behavior, sexual orientation/gender identity, motivation to move out, and family demographics. Sexual orientation/gender identity was not associated with youth delinquent behavior after controlling for covariates. Being motivated to move out moderated the association between bullying victimization and delinquent behavior. Sexual orientation/gender identity and being motivated to move out of low-resourced communities jointly contributed to the moderating effect between bullying victimization and delinquent behavior. For non-heterosexual/cisgender youth, bullying victimization is correlated with increased delinquent behavior for those with low motivation to move out of their communities compared with those with an average or higher level of motivation to stay. However, such a moderating effect was not shown for heterosexual youth.

## 1. Introduction

Bullying victimization, which is defined as physical, verbal, or psychological abuse and happens within or near a school [1], is a serious problem for adolescents in general; for racial and sexual minorities, bullying victimization is especially concerning. According to the GLSEN School Climate Survey, in 2019, 25.7% of non-heterosexual/cisgender adolescents reported being physically assaulted, 68.7% reported being verbally harassed, 58.3% reported being sexually harassed, and 59.1% reported feeling unsafe in their school [2]. Empirical research findings have also indicated that African American adolescents are at significant risk of being bullied by their peers [3,4] because of anti-Black racism [5]. One study found that bullying victimization was highest among African American adolescents relative to the youth of other racial and ethnic groups [6]. In addition, African American youth who identify as sexual minorities might experience homophobic harassment or aggression and racial and ethnic slurs [7], placing them at greater risks for bullying victimization [8].

According to Minority Stress Theory, there is an array of stressors related to being a part of a minoritized group [9], and minorities’ mental health likely is impacted by the extent to which their social environment stigmatizes their identity [10]. For racial and sexual minorities, stigmatization of being a minority itself can result in bullying victimization, which can negatively impact behavioral health and psychosocial wellbeing [11]. One of the most consistent findings in research on bullying is the relationship between victimization and delinquent behavior, which is not only supported empirically but also theoretically. According to Higgins et al.’s [12] study with a sample of 725 African American youth, victims of bullying show delinquent trajectories. Park and Metcalfe’s [13] study, which comprised a national, longitudinal sample of South Korean adolescents, showed that bullying victimization was positively correlated with delinquent behavior, and bullying victimization over consecutive years predicted analogous behavior, violence, and theft. Findings from Glassner and Cho’s [14] study also suggest that childhood bullying victimization correlates with substance use risk in adolescence and young adulthood for males. Additionally, McCuddy and Esbensen [15], which relied on data on middle school students in the Gang Resistance Education and Training program, found that victims of cyberbullying exhibited a higher propensity for non-violent delinquent behavior and substance use.

Theories, such as Agnew’s [16] General Strain Theory (GST) and Cohen and Felson’s [17] Routine Activities Theory (RAT), have often been used to inform how bullying victimization might be related to delinquent behavior. Some researchers have posited that what is termed delinquent behavior might be a manifestation of stress, trauma, or a cry for help or attempts to cope [18]. The GST views victimization as a significant source of strain, which most likely reinforces delinquent conduct [19]. RAT argues that victimization results from the convergence of a suitable target, motivated offender, and a lack of capable guardianship [17]. The issues of opportunity or lifestyle can place youth at the highest risk of bullying victimization, which may subsequently correlate to delinquent behavior. Understanding the linkage between bullying victimization and delinquent behavior among African American adolescents is critical given their disproportionate experiences in victimization and offending in the United States [12].

Although a strong association between bullying victimization and delinquent behavior has been documented, it is important to also recognize that not all victims of bullying are at risk of displaying delinquent behavior. For adolescents in urban schools, identifying influential factors and developing resilience that potentially attenuates the linkage between victimization and adverse outcomes is especially critical [20] as these adolescents tend to have fewer resources than their suburban peers in helping them overcome adversities. Researchers on African American and non-heterosexual/cisgender adolescents have also come to recognize the salience of understanding influential factors that can help these youth to cope with adversities, such as chronic victimization, in healthier ways [21,22,23]. One influential factor, which has yet been explored to our knowledge, is adolescents’ motivation to move out of their community to escape poverty and violence. Overcoming poverty and achieving upward mobility is a difficult feat for African American adolescents in urban neighborhoods [24] as they are confronted with structural barriers, poverty, and racism triggered violence, which impedes their future goals [20]. However, as literature on adolescent future orientation (i.e., an individual’s thoughts, beliefs, plans, and hopes for the future) suggests, adolescents with a positive outlook of their future are less apt to display problematic behavior or engage in risky behavior that would otherwise jeopardize their future goals [25,26,27]. Conceivably, adolescents who are doing what they can to leave their community are likely to be future-oriented, and similar to positive future orientation, feeling motivated to leave the community would likely buffer the positive association between bullying victimization and delinquent behavior.

The present study explores whether motivation to move out of low-resourced communities might moderate the relationship between bullying victimization and delinquent behavior among heterosexual and non-heterosexual/cisgender African American adolescents on Chicago’s Southside. We focus specifically on these two groups for the following reasons. Research on bullying victimization among urban African American youth is limited, albeit emerging. However, there remains a serious dearth of empirical studies on urban African Americans who identify as non-heterosexual/cisgender and their experiences of bullying victimization. Given their dual minoritized status (i.e., minority and sexual orientation), their likelihood of being victimized by their peers is elevated. Due to the presence of racism and homophobia in their social environments, non-heterosexual/cisgender African American adolescents may lack the needed social supports from their family, peers, and adult authority figures (e.g., teachers). The focus of this study contributes to illuminating resiliency factors for vulnerable African American youth in low-resourced communities.

### The Current Study

Building upon extant research, the current study used a sample of African American adolescents to examine how being motivated to move out of a disadvantaged community may moderate the association between bullying victimization and delinquent behavior and whether this differs between heterosexual youth and non-heterosexual/cisgender youth. We specifically addressed the following question: (a) Are adolescents’ sexual orientation/gender identity and motivation to move out of a disadvantaged community associated with their delinquent behavior when bullying victimization and other factors are accounted for? We also propose and test the following hypotheses: (a) Being motivated to move out of a disadvantaged community will buffer the positive association between bullying victimization and delinquent behavior; (b) being motivated to move out of a disadvantaged community as a moderator of the association between bullying victimization and delinquent behavior will be stronger for non-heterosexual/cisgender youth than heterosexual youth.

## 2. Materials and Methods

### 2.1. Participants

Data from this study were drawn from the Resilience Project, which investigated risk and protective factors associated with behaviors among African American youth in multiple low-income neighborhoods in Chicago’s Southside. Study participants were recruited from three high schools, one youth church group, two community youth programs, and four public venues (e.g., parks, movie theatre) from Chicago’s Southside low-income communities. The number of individuals approached at each site and the individuals who enrolled in the study were as follows: schools (606/579), community youth programs (42/38), churches (49/44), and public venues (56/39). An overall response rate of 87% was achieved.

### 2.2. Procedures

Flyers with information about the study were posted at the high schools, youth church groups, community youth programs, and public venues. The school principals as well as leaders of a church group and community youth program provided permission for the researchers to recruit study participants. Trained research assistants first introduced the study to all potential study participants with a detailed letter along with parental consent forms. To be eligible for the study, participants had to self-identify as African American. They were between the ages of 13 and 24, which represents early to late adolescence. Underage adolescents who returned consent forms signed by their parent or guardian and provided assent were enrolled in the study. Adolescents who were 18 years old and older provided informed consent. Participants in schools, youth community programs, and church groups were given a questionnaire at their respective locations. Participants in public venues were provided a questionnaire in quiet spaces at or near those venues. Questionnaires were only given if a parent or a guardian was present to provide consent. The questionnaire took approximately 45 min to complete, and upon the completion of the questionnaire, participants were given USD 10. A total of 655 youth participated in the study. The missing data were approximately 5% for a study variable. After excluding cases with missing values, 541 cases with complete data were included in the study. The study was approved by the University Institutional Review Board of the last author.

### 2.3. Measures

*Delinquent behavior* was conceptualized as a dependent variable in the multivariate models. The survey included a statement, “Now we are going to ask you some questions about your behavior within the last 12 months. In the past months, how often have you done the following,” and was followed with ten questions related to delinquent behavior. For this study, three questions asking the frequency of engaging in delinquent behavior, such as “tak[ing] something not belonging to you worth under $50,” “tak[ing] something from a store without paying for it,” and “tak[ing] an expensive part of a car without the permission of the owner (for example, radio, tire, rims),” were selected. Response options are 0 times (0), 1–2 times (1), 3–5 times (2), 6–8 times (3), 9–11 times (4), and 12 or more times (5). The scores across items were summed up to form a scale with larger values indicating more frequent delinquent behavior. The maximum value was capped at 20 because of the rarity of larger values (α = 0.88). Items for this variable were used in prior studies [28,29].

*Bullying victimization* was conceptualized as the independent variable in the multivariate models and was derived from the University of Illinois Victimization Scale [30]. The survey included a statement, “For each of the following questions, choose how many times you did this activity or how many times these things happened to you in the last 30 days,” which was followed by eighteen questions related to peer relationships. For this study, four items that measure bullying victimization were chosen, including “other students picked on me,” “other students made fun of me,” “other students called me names,” and “I got hit and pushed by other students.” Response options are 0 times (0), 1–2 times (1), 3–5 times (2), 6–8 times (3), 9–11 times (4), and 12 or more times (5). The scores across items were averaged to form a scale with larger values indicating more intense bullying victimization (α = 0.86).

*Sexual orientation/gender identity* was conceptualized as a moderating variable. Youth were asked, “How do you identify yourself?” Response options are “heterosexual (you are sexually attracted to the opposite sex),” “Homosexual (you are sexually attracted to the same sex),” “Bisexual (you are sexually attracted to both sexes),” “Transgender (you identify as another gender than the gender you grew up as),” “Pansexual (you are sexually attracted to people of all gender identities and biological sexes),” and “Other”. Youth who identified themselves as heterosexual were coded as 1, otherwise, they were coded as 0 as non-heterosexual/cisgender.

*Motivation to move out of the community* was also conceptualized as a moderator. The survey included a statement, “Think of all the stressful things you just reported in about your community. For each item below, circle one answer that best matches the way your (sic) try to manage or deal with such violence,” which was followed with 29 questions related to responses to community violence. The present study selected three questions, which were derived from the Coping with Community Violence Scale [31], including the following: “I try to work hard in an activity that may help me to get out of my community,” “I try to work hard in school so that I can get out of my community,” and “I work to save money so that I can get out of my community.” Response options are never (1), sometimes (2), often (3), and very often (4). The responses were averaged to form a scale with higher values indicating a stronger motive of moving out of the community (α = 0.71).

Multiple covariates were included in the multivariate models to partial out potential confounding influence. These included *age* (12 to 18; a few cases of 19 were recoded into 18), *sex* (0 = male, 1 = female), *ever using a drug* (the survey included a statement, “Now we are going to ask you some questions about adolescent behaviors that may or may not apply to you. Circle only one answer,” followed by six questions: “Have you ever smoked a whole cigarette or cigar (tobacco)?”, “Have you ever taken ecstasy (Molly, MDMA)?”, “Have you ever used Lean or Krokodil (cough syrup, codeine)?”, Have you ever had at least one drink of alcohol?”, “Have you ever used marijuana (blunts, pot, weed)?”, and “Have you ever used crack or cocaine?”; 0 = no; 1 = yes), *ever involved in the juvenile justice system* (0 = no; 1 = yes), and *family structure* (1 = two-parent family; 2 = single-parent family; 3 = other). *Receiving public assistance* (0 = no; 1 = yes) was measured with a question which asked youth whether they were currently receiving free or reduced lunch and/or SNAP benefits (Link Card).

### 2.4. Analysis Plan

Bivariate analyses were first conducted to describe the overall sample and compare the difference between heterosexual and non-heterosexual/cisgender youth across study variables. Multivariate models were then used to evaluate how bullying victimization, sexual orientation/gender identity, and being motivated to move out of the community were associated with youth delinquent behavior after controlling for covariates, and how sexual orientation/gender identity and being motivated to move out of the community may moderate the association between bullying victimization and youth delinquent behavior. Due to the fact that the majority of the youth did not report delinquent behavior and the number of delinquent acts was highly skewed, negative binomial regression was used in the multivariate modeling. Negative binomial regression is similar to regular multiple regression, but it is suitable for modeling a count dependent variable with a dispersed distribution. Negative binomial regression is a generalization of Poisson regression, but it loosens the restrictive assumption that the variance is equal to the mean made by the Poisson model. We first conducted multivariate modeling based on the entire sample to detect the interaction that indicates a moderating effect among heterosexuality, bullying victimization, and moving motivation, and then conducted supplementary subgroup analyses based on youth sexual orientation/gender identity separately to illustrate the difference between these two groups. In each stage, we first used one model to examine the associations without considering the moderating effect and another model that accounted for the moderating effect. Finally, we computed the moderating effects of being motivated to move out of the community on bullying victimization and delinquent behavior by sexual orientation/gender identity. Analyses were performed with Stata 15 (StataCorp LLC, College Station, TX, USA).

## 3. Results

Table 1 presents youth characteristics including age (*M* = 15.85 years old, *SD* = 1.37), sex (55% of female), ever using a drug (60%), and ever being involved in the juvenile justice system (11%). In addition, youth reported the intensity of bullying victimization in the last 30 days (*M* = 0.55, *SD* = 0.83), delinquent behavior (*M* = 1.65, *SD* = 3.64), and intensity of being motivated to move out of the community (*M* = 1.76, *SD* = 0.84). Most adolescents were in a single-parent family (57%), one-third in a two-parent family, and the remaining (10%) were in another family status. Nearly three-quarters (72%) of the youth were receiving some type of public assistance.

Compared with heterosexual youth (*n* = 450), non-heterosexual/cisgender youth (*n* = 91) were more likely to be female, have used a drug, be involved in the juvenile justice system, experience more intense bullying victimization, and have a lower motivation to move out of the community. However, they were not statistically different from each other in other aspects including age, delinquent behavior, family structure, and receiving public assistance (see Table 1).

## 4. Multivariate Model Results

### 4.1. Results Based on the Total Sample 

As shown in Table 2, model 1 presents results based on the total sample without considering the moderating effects, while model 2 includes interaction terms to assess sexual orientation/gender identity and motivation to move out of the community’s moderating effect on the association between bullying victimization and youth delinquent behavior. The alpha (α) in the model is the dispersion parameter that assesses whether a negative binomial model is more suitable than a Poisson model which models count dependent variables based on the Poisson distribution. If the alpha is significantly greater than 0, it indicates that the data are over-dispersed, and a negative binomial model has a better fit than a Poisson model. The alpha for both models is significantly greater than 0 (b = 2.94 to 3.07; *p* < 0.001) and justifies the adoption of negative binomial regression. When interaction terms are included in the model, the pseudo-*r*^2^ increases from 0.06 to 0.07, indicating that the model accounting for the moderating effects has a better fit.

Model 1 in Table 2 shows that sexual orientation/gender identity were not associated with youth delinquent behavior after controlling for covariates (incident relative risk (IRR) = 0.72, 95% confidence interval (95% CI) = 0.44, 1.18). Older youth (IRR = 1.15, 95% CI = 1, 1.31), ever using a drug (IRR = 2.36, 95% CI = 1.61, 3.47), ever involved in the juvenile justice system (IRR = 2.61, 95% CI = 1.5, 4.53), and more intense bullying victimization (IRR = 1.81, 95% CI = 1.44, 2.28) were associated with more delinquent behavior. On the other hand, being female (IRR = 0.62, 95% CI = 0.43, 0.92) and having stronger motivation to move out of the community (IRR = 0.74, 95% CI = 0.58, 0.93) were associated with less delinquent behavior.

Model 2 in Table 2 shows that motivation to move out of the community had a moderating effect on the association between bullying victimization and delinquent behavior (IRR = 0.46, 95% CI = 0.23, 0.95) and that sexual orientation/gender identity and being motivated to move out of the community may jointly contribute to the moderating effect (IRR = 2.06, 95% CI = 0.92, 4.61). The subgroup analysis based on heterosexual and non-heterosexual/cisgender youth as well as the effect visualization below can further illustrate this relationship.

### 4.2. Results Based on Subgroup Analyses

Models 1 and 2 in Table 3 show the results of negative binomial models based on non-heterosexual/cisgender youth. The dispersion parameter indicates that a negative binomial model is a better option than a Poisson model (b = 2.02 to 2.39; *p* < 0.001). Model 1 shows that youth involved in the juvenile justice system (IRR = 4.61, 95% CI = 1.66, 12.82) and bullying victimization (IRR = 2.09, 95% CI = 1.32, 3.31) were risk factors for youth delinquent behavior. Model 2 shows that motivation to move out of the community had a moderating effect on bullying victimization and delinquent behavior (IRR = 0.43, 95% CI = 0.23, 0.82). The pseudo- *r*^2^ increases from 0.08 for the model without the moderating effect to 0.1 for the model accounting for the moderating effect, indicating the latter has a better fit.

Models 1 and 2 in Table 4 show the results of negative binomial models based on heterosexual youth. The dispersion parameter indicates that a negative binomial model is a better option than a Poisson model (b = 2.98; *p* < 0.001). Model 1 shows that older youth (IRR = 1.22, 95% CI = 1.05, 1.41), youth who ever used a drug (IRR = 3.12, 95% CI = 2.05, 4.74), and youth who were ever involved in the juvenile justice system (IRR = 2.54, 95% CI = 1.33, 4.85) and experienced more frequent bullying victimization (IRR = 1.93, 95% CI = 1.46, 2.56) were associated with more delinquent behavior. Model 2 shows that motivation to move out of the community did not have a moderating effect on bullying victimization and delinquent behavior (IRR = 0.93, 95% CI = 0.64, 1.34). As a result, the pseudo- *r*^2^ for the models without and with the moderating effect is the same, suggesting that the moderating effect is negligible.

### 4.3. Moderating Effect Illustration

**Figure 1:**
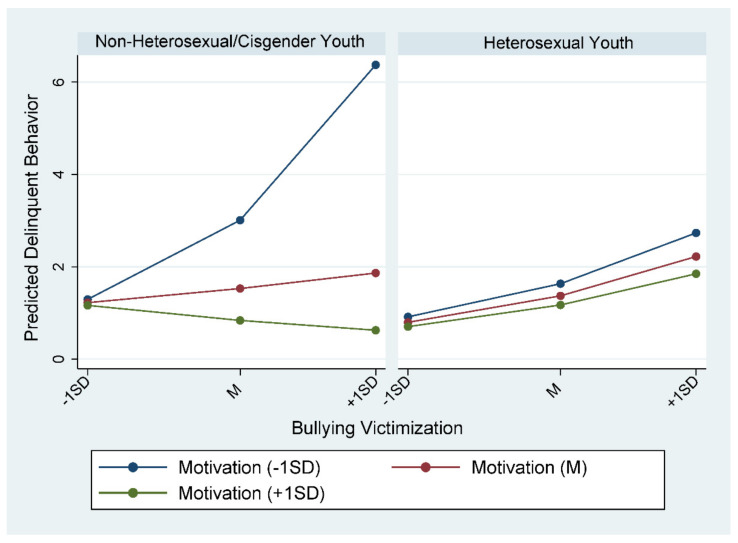
The influence of motivation to move on the association between bullying victimization and delinquent behavior by sexual orientation/gender identity.

Figure 1 illustrates sexual orientation/gender identity and motivation to move out of the community’s moderating effects on bullying victimization and youth delinquent behavior. The left panel in Figure 1 shows the moderating effect of motivation to move out of the community on bullying victimization and delinquent behavior for non-heterosexual/cisgender youth, while the right panel is for heterosexual youth. For non-heterosexual/cisgender youth, when bullying victimization frequency was low (one standard deviation [SD] below the mean), the predicted delinquent behavior was slightly more than 1 regardless of youth motivation to move out of the community; however, when the bullying victimization frequency increased to a high level (one SD above the mean), the predicted delinquent behavior increased to over 6 for those who had a low level of motivation to move, increased to about 2 for those who had average motivation to move, and declined to less than 1 for those who had a high level of motivation to move. In contrast, such a moderating effect is not shown for heterosexual youth. When bullying victimization frequency increased, there was a similarly moderate increase in delinquent behavior among youth with different levels of motivation to move out of the community.

## 5. Discussion

The current study aimed to explore how African American youth’s motivation to move out of disadvantaged communities might buffer the relationship between bullying victimization and delinquent behavior, and whether the relationship differs for heterosexual youth versus those who are non-heterosexual/cisgender. Concerning the first research question, our findings showed that sexual orientation/gender identity was not associated with delinquent behavior although older age, drug use, involvement in the juvenile justice system, and bullying victimization were. These findings were consistent with the extant research literature, which showed that older age, using drugs, prior involvement in the juvenile justice system, and being a victim of bullying are important predictors of adolescents’ delinquent behavior [32,33,34,35,36], which hold for both heterosexual and non-heterosexual/cisgender African American youth. This finding also supports the propositions made by GST, which maintains that strains (e.g., bullying victimization) increase the odds of delinquent behavior [16,18], and the Minority Stress Theory, which proposes that difficult social situations (e.g., bullying victimization) are likely to reinforce stress for minorities, resulting in health and behavioral problems [9].

Regarding the proposed research question, our results showed that motivation to move out of disadvantaged communities buffered the relationship between bullying victimization and delinquent behavior for non-heterosexual/cisgender youth. Although studies, to date, have not examined whether motivation to move out of the community is a potential buffer, it appears to be for the non-heterosexual/cisgender sample in our study. African American adolescents in low-resourced urban neighborhoods such as on Chicago’s Southside are consistently exposed to violence, such as bullying victimization, which likely has an impact on their behaviors, including delinquent behavior [33,35]. As studies have documented, non-heterosexual/cisgender adolescents are especially prone to witnessing and experiencing violence in their schools and their communities [2,36]. For non-heterosexual/cisgender adolescents living in low-resourced urban neighborhoods, support and resources for lesbian, gay, bisexual, transgender, and queer individuals tend to be limited, as a result they might perceive escaping their community as the only solution. Consequently, they are less likely to exhibit delinquent behavior even when confronted with challenges, such as bullying victimization. They might be more inclined to refrain from delinquent behavior, which could get them into trouble and make it more difficult to move out of their community. Perhaps these adolescents tend to play by the rules, which can increase their odds of moving out of their community.

Addressing the first hypothesis, motivation to move out of the community was found to have a moderating effect on bullying victimization and delinquent behavior for non-heterosexual/cisgender youth but not for heterosexual youth, although bullying victimization is positively associated with delinquent behavior for both groups as shown in the literature [33,35]. As previously stated, motivation to move out of the community may be a salient protective factor for non-heterosexual/cisgender adolescents in low-resourced urban communities, especially where social supports from parents, teachers, and other adult figures that can buffer negative psychosocial outcomes of bullying victimization might be lacking. In addition, despite gay-related stressful life events frequently experienced by non-heterosexual/cisgender adolescents in urban communities, personal resources such as higher self-esteem might be inadequate in coping with stress. For instance, an earlier study by Rosario et al. [37] examined the relationships between gay-related and non-gay-related stressful life events, self-esteem, emotional distress, and multiple problem behaviors of Hispanic and African American gay and bisexual youth in a large urban area. The study found that higher self-esteem was not a protective buffer of the association between gay-related stressful life events and emotional distress or between gay-related stressful life events and a multiple of problem behaviors. Non-heterosexual/cisgender adolescents who are motivated to move out of their community may do what they can and abstain from any conduct that would jeopardize their possibility of moving out. Moreover, identifying protective factors for non-heterosexual/cisgender youth is particularly important, especially given that non-heterosexual/cisgender youth are twice as likely as their heterosexual peers to be involved in the juvenile justice system for non-violent offenses [38].

### 5.1. Limitations and Implications for Research

The present study is not without important limitations. First, this study examines non-heterosexual/cisgender and heterosexual African American youth who live in Chicago’s Southside. This is a very unique population and limitations exist around generalizing our findings outside of this sample of youth. Moreover, reports of bullying victimization and delinquent behavior were self-reported measures. These measures allow for the potential of self-report bias to inflate associations between study variables. Similarly, the respondents were given USD 10 for their participation, which might have introduced response bias. Furthermore, the cross-sectional design precludes any assumptions of temporal relationships among variables. In terms of adolescent development, some adolescents may not have experienced any sexual attraction and responded as “Other.” In addition, measures for motivations to move out of the community and delinquent behavior are limited. Future research might consider additional measures for motivations to move out of the community, such as being motivated to move out for a better future. Likewise, delinquent behavior should be measured more extensively with other forms of delinquency, such as violent crimes. Finally, related to sexual orientation/gender identity, there was a significant disproportion between non-heterosexual/cisgender and heterosexual samples in our study, which may reduce the comparability of the two groups. In subgroup analysis, the number of subjects for non-heterosexual/cisgender youth was small, which may further limit the model’s statistical power in detecting potential associations between the predictors and the dependent variable. However, the statistical significance of the interaction regardless of the small sample suggests motivation to move out of the community is a robust moderator on bullying victimization’s association with delinquent behavior for these youth.

These limitations notwithstanding, the present study highlights significant differences in bullying experiences and motivations to leave their communities between non-heterosexual/cisgender and heterosexual African American youth. Identification of motivations to leave current communities as a moderator for non-heterosexual/cisgender African American youth is significant because it shows the importance of the environment in which youth live as a protective factor. In order to engage youth in bullying intervention/prevention programs, it is important to understand the contexts in which youth develop. Therefore, acknowledging and discussing supports in their communities or the lack of supports that exist in their community may be efficacious. Future research could provide a deeper investigation of the motivation behind why non-heterosexual/cisgender youth want to leave their communities to shine a light on the different motivations that youth may have. Furthermore, it is important to consider whether the motivation to leave the community would be a protective buffer among adolescents who have no intention of leaving their community.

Future researchers also may wish to examine differences within groups of non-heterosexual/cisgender African American youths; additional marginalized identities may contribute to the associations examined in this study. For example, an intersectional perspective suggests that variation may be explained by neighborhood-level factors or other intersecting identities (e.g., religious/spiritual beliefs, ethnicity, and culture, ability, gender identity, etc.). Additionally, the age at which adolescents “come out” or disclose their sexual orientation/gender identity may be an important future consideration; some youth may experience parental or peer rejection, conflict within the household, or victimization that targets one’s sexual orientation/gender identity or expression earlier than youth who choose to not disclose their sexual orientation/gender identity until later in adolescence or adulthood [39].

### 5.2. Implications for Theory and Practice

Youth living with dual-marginalized identities are at a higher risk for experiencing bullying victimization, which potentially increases their risk of engaging in delinquent activities. This relationship has been supported by several theoretical frameworks, such as GST and RAT. However, these theoretical models must be further tested with a sample of non-heterosexual/cisgender adolescents. Findings from the study also are consistent with the Minority Stress Theory, which suggests that the stigma of belonging to minoritized groups [9,10] can contribute to bullying victimization. The Minority Stress Theory has implications for practice, as it can guide practitioners in thoroughly assessing for prejudice, stigma, and homophobia.

Despite researchers documenting these disparate rates and although intervention research is growing in this area, most bullying prevention and intervention programs still do not explicitly address stigma, issues of diversity, stereotypes, prejudice, and discrimination. Furthermore, they do not typically assess the impact of stigma-based bullying or bullying among racial, ethnic, and/or non-heterosexual/cisgender adolescents. More programs focus on bullying prevention related to non-heterosexual/cisgender-related and disability-related bullying; and a lesser amount focus on reducing bullying experienced by racial and ethnic minority adolescents [40]. More research is necessary to develop, expand, and evaluate bullying prevention programs to be more inclusive of intersecting identities.

Additionally, it is important to strategize about the best methods of enhancing bullying prevention and intervention programs. Interventions that target stigma related to only one identity (e.g., race) may not reduce bullying experienced by an adolescent who identifies as a racial minority and a sexual or gender minority. Interventions need to take an intersectional perspective so that multiple minority-identity adolescents are better supported and included in bullying prevention efforts.

The current study highlighted the role of motivation to move out of one’s community, which includes aspects of having a future orientation and upward mobility, as a protective factor in the association between bullying victimization and delinquent behavior among non-heterosexual/cisgender, African American adolescents living in an economically disadvantaged urban area. This study adds to a body of studies highlighting potential factors to strengthen, bolster, or include in prevention programming. Future studies may wish to examine this motivation to move out of one’s community in relation to other protective factors, such as the ability to respond nonviolently to conflict, parenting behaviors, family and peer relationships, community connections, and positive school climate variables [41]. Additionally, researchers may wish to examine this motivation in relation to assessments of upward mobility and future orientation in order to observe how together these processes may impact outcomes among victimized youth who identify as African American and non-heterosexual/cisgender.

## 6. Conclusions

Adolescents with minority status or multiple minority statuses, such as being non-heterosexual/cisgender and African American, are frequent targets of bullying victimization by their peers. Bullying and other violence constitute adverse childhood experiences taking a toll observed in behavior and on mental health [42]. The current study examined the concept of motivation to move out of Chicago’s Southside as a moderator of bullying victimization and delinquent behavior among these youth. This is particularly important as research notes that adolescents may engage in acts of delinquent behavior after bullying victimization. Interrupting that behavioral response is vital for healthy adult development. To our knowledge, adolescents’ motivation to move out of their community to escape poverty and violence has not been explored. In order to eliminate victimization, participants actively saved money and adopted other measures to create a better future for themselves. Moving out of the community was observed as a viable means. However, although females suffered greater victimization, they expressed less motivation to leave the community. This study emphasizes the need to support motivation toward upward mobility as a protective factor for non-heterosexual/cisgender adolescents.

## Figures and Tables

**Table 1:** Sample characteristics.

Variable	Total (*n* = 541)		Non-Heterosexual/ Cisgender (*n* = 91)		Heterosexual (*n* = 450)		*p ^a^*	Range	α
	*M* or %	*SD*	*M* or %	*SD*	*M* or %	*SD*			
Age	15.85	1.37	15.77	1.31	15.87	1.38	0.535	12 to 18	
Female	0.55		0.84		0.49		<0.001	1, 2	
Ever using a drug (yes)	0.6		0.77		0.57		<0.001	0, 1	
Ever involved in juvenile justice system (yes)	0.11		0.21		0.09		<0.001	0, 1	
Bullying victimization	0.55	0.83	0.77	0.98	0.51	0.79	0.007	0 to 4	0.86
Delinquent behavior	1.65	3.64	2.57	4.8	1.47	3.33	0.08	0 to 20	0.88
Motivation to move out of the community	1.76	0.84	1.59	0.88	1.8	0.83	0.035	0 to 3	0.71
Family structure							0.08		
Two-parent	0.33		0.29		0.34			1	
Single-parent	0.57		0.55		0.57			2	
Other	0.1		0.16		0.09			3	
Receiving public assistance	0.72		0.69		0.73		0.451	0, 1	

*Note*: *^a^* For group comparisons, Chi-square tests were used for the categorical variables, and *t*-tests were used for numeric variables.

**Table 2:** A negative binomial model associated with delinquent behavior of total youth (*n* = 541).

Variable	Model 1				Model 2			
	IRR	95% CI	*p*		IRR	95% CI	*p*	
Heterosexual (yes vs. no)	0.72	(0.44, 1.18)	0.193		0.58	(0.14, 2.34)	0.443	
Age	1.15	(1, 1.31)	0.044	**	1.16	(1.01, 1.33)	0.03	**
Female (vs. male)	0.62	(0.43, 0.92)	0.016	**	0.66	(0.45, 0.97)	0.034	**
Ever using a drug (yes vs. no)	2.36	(1.61, 3.47)	<0.001	***	2.6	(1.76, 3.83)	<0.001	***
Ever involved in the juvenile justice system (yes vs. no)	2.61	(1.5, 4.53)	0.001	***	2.59	(1.49, 4.5)	0.001	***
Bullying victimization	1.81	(1.44, 2.28)	<0.001	***	5.1	(1.52, 17.12)	0.008	***
Motivation to move out of the community	0.74	(0.58, 0.93)	0.011	**	0.75	(0.37, 1.51)	0.415	
Family structure (two-parent)								
Single parent	1.3	(0.88, 1.92)	0.185		1.3	(0.89, 1.92)	0.177	
Other	1.18	(0.62, 2.24)	0.612		1.2	(0.64, 2.25)	0.573	
Receiving public assistance	0.88	(0.59, 1.34)	0.559		0.84	(0.55, 1.29)	0.429	
Moderators								
Heterosexual × bullying					0.39	(0.09, 1.65)	0.201	
Heterosexual × Motivation to move out of the community					1.13	(0.53, 2.41)	0.749	
Bullying × Motivation to move out of the community					0.46	(0.23, 0.95)	0.035	**
Heterosexual × Bullying × Motivation to move out of the community					2.06	(0.92, 4.61)	0.078	*
α	3.07	(2.47, 3.81)	<0.001	***	2.94	(2.36, 3.66)	<0.001	***
Pseudo-*r*^2^	0.06				0.07			
Log-likelihood	−771.67				−767			

* *p* < 0.1; ** *p* < 0.05; *** *p* < 0.01.

**Table 3:** A negative binomial model associated with delinquent behavior of non-heterosexual/cisgender youth (*n* = 91).

Variable	Model 1					Model 2		
	IRR	95% CI	*p*		IRR	95% CI	*p*	
Age	0.95	(0.66, 1.36)	0.769		0.96	(0.68, 1.35)	0.806	
Female (vs. male)	0.39	(0.14, 1.11)	0.077	*	0.33	(0.12, 0.92)	0.033	**
Ever using a drug (yes vs. no)	0.73	(0.26, 2.01)	0.54		0.79	(0.29, 2.17)	0.651	
Ever involved in the juvenile justice system (yes vs. no)	4.61	(1.66, 12.82)	0.003	***	4.23	(1.6, 11.22)	0.004	***
Bullying victimization	2.09	(1.32, 3.31)	0.002	***	7.31	(2.48, 21.49)	<0.001	***
Motivation to move out of the community	0.65	(0.38, 1.11)	0.112		1.13	(0.6, 2.11)	0.71	
Family structure (two-parent)								
Single parent	1.09	(0.43, 2.76)	0.86		1.08	(0.45, 2.57)	0.863	
Other	0.6	(0.18, 2.07)	0.423		0.7	(0.22, 2.25)	0.546	
Receiving public assistance	0.52	(0.21, 1.29)	0.156		0.37	(0.15, 0.91)	0.031	**
Moderators								
Bullying × Motivation to move out of the community					0.43	(0.23, 0.82)	0.01	***
α	2.39	(1.47, 3.9)	<0.001	***	2.02	(1.2, 3.38)	<0.001	***
Pseudo-*r*^2^	0.08				0.1			
Log-likelihood	−155				151.56			

*p* < 0.1 *; *p* < 0.05 **; *p* < 0.01 ***.

**Table 4:** A negative binomial model associated with delinquent behavior of heterosexual youth (*n* = 450).

Variable	Model 1				Model 2			
	IRR	95% CI	*p*		IRR	95% CI	*p*	
Age	1.22	(1.05, 1.41)	0.008	***	1.22	(1.06, 1.41)	0.007	***
Female (vs. male)	0.67	(0.44, 1.02)	0.061	*	0.68	(0.45, 1.03)	0.067	*
Ever using a drug (yes vs. no)	3.12	(2.05, 4.74)	<0.001	***	3.13	(2.06, 4.76)	<0.001	***
Ever involved in the juvenile justice system (yes vs. no)	2.54	(1.33, 4.85)	0.005	***	2.5	(1.3, 4.79)	0.006	***
Bullying victimization	1.93	(1.46, 2.56)	<0.001	***	2.24	(1.04, 4.85)	0.04	**
Motivation to move out of the community	0.82	(0.63, 1.06)	0.136		0.85	(0.63, 1.15)	0.284	
Family structure (two-parent)								
Single parent	1.18	(0.77, 1.82)	0.448		1.19	(0.77, 1.84)	0.427	
Other	1.36	(0.66, 2.83)	0.408		1.37	(0.66, 2.85)	0.401	
Receiving public assistance	1.02	(0.63, 1.65)	0.932		1.04	(0.64, 1.68)	0.886	
Moderators								
Bullying × Motivation to move out of the community					0.93	(0.64, 1.34)	0.683	
α	2.98	(2.33, 3.82)	<0.001	***	2.98	(2.33, 3.81)	<0.001	***
Pseudo-*r*^2^	0.07				0.07			
Log likelihood	−607.3				−607.2			

*p* < 0.1 *; *p* < 0.05 **; *p* < 0.01 ***.

## Data Availability

The study did not report any data.
